# Examination of Ligand-Dependent Coactivator Recruitment by Peroxisome Proliferator-Activated Receptor-α (PPARα)

**DOI:** 10.1155/PPAR/2006/69612

**Published:** 2006-06-27

**Authors:** Eric S. Tien, Daniel B. Hannon, Jerry T. Thompson, John P. Vanden Heuvel

**Affiliations:** ^1^Department of Veterinary and Biomedical Sciences and Center for Molecular Toxicology and Carcinogenesis, Pennsylvania State University, 201 Life Sciences Building, University Park, PA 16802, USA; ^2^NIEHS, MD E4-07, PO Box 12233, Research Triangle Park, NC 27709, USA

## Abstract

The ligand-dependent recruitment of coactivators to peroxisome proliferator-activated receptor-α (PPARα) was examined. PPAR-binding protein (PBP), PPARγ coactivator-1α (PGC-1α), steroid receptor coactivator-1 (SRC-1), and CBP/p300-interacting transactivator with ED-rich tail 2 (CITED2) affected PPARα activity in the presence of Wy-14,643. The effects on PPARα activity in light of increased or decreased expression of these coactivators were qualitatively different depending on the ligand examined. Diminished expression of PGC-1α, SRC-1, or PBP by RNAi plasmids affected natural or synthetic agonist activity whereas only Wy-14,643 was affected by decreased PGC-1α. The interaction of PPARα with an LXXLL-containing peptide library showed ligand-specific patterns, indicative of differences in conformational change. The association of coactivators to PPARα occurs predominantly via the carboxyl-terminus and mutating ^456^LHPLL to ^456^LHPAA resulted in a dominant-negative construct. This research confirms that coactivator recruitment to PPARα is ligand-dependent and that selective receptor modulators (SRMs) of this important protein are likely.

## INTRODUCTION

The peroxisome proliferator-activated receptors (PPARs) are a subfamily of nuclear receptors (NRs) that contains three members (PPARα, β, γ). These receptors function in a wide array of metabolic processes including fatty acid and lipid homeostasis, adipocyte differentiation, and control of cell growth and proliferation [1–3]. PPARα responds to a class of chemicals called peroxisome proliferators (PPs). These chemicals were so named for the initial finding that they caused increases in peroxisome number and size with prolonged treatment. Some PPARα ligands promote tumor formation in rodents [4, 5]. Paradoxically, PPARγ ligands have anticarcinogenic properties in a variety of tissues [6]. An example of these compounds is conjugated linoleic acid (CLA), which is also a ligand for PPARα.

The regulation of gene expression by nuclear receptors is dependent on the recruitment of accessory proteins to the transcriptional complex. Coregulators are a class of proteins that function to enhance or repress the activity of the transcriptional complex around the nuclear receptor dimer. Coactivators were originally found to contain a specific sequence of amino acids (LXXLL where X is any amino acid) that was termed the NR box or receptor interacting domain (RID). Many coactivators contain one or more of these motifs and use these domains to interact with nuclear receptors [7, 8]. Though not all coactivators utilize LXXLL to interact with different nuclear receptors (such as steroid receptor RNA activator (SRA) and the CBP/p300-interacting transactivator with ED-rich tail (CITED) family of proteins [9, 10]), many studies have used LXXLL as a target sequence to identify more nuclear receptor interacting proteins.

One of the more interesting aspects of the PPAR family of nuclear receptors is the size of the ligand-binding pocket. The PPARs have one of the largest ligand-binding domains by volume of all known nuclear receptors at over 1300 Å^3^ [11]. By comparison, the ligand-binding pocket of the liver X receptor (LXR) is 830 Å^3^ which is a more typical volume for nuclear receptors [12]. The size of the ligand-binding pocket allows the PPARs to accommodate a very wide range of ligand shapes and sizes. Known ligands for the PPARs include hypolipidemic drugs (fibrates), fatty acids (CLA), and plasticizers (phthalates) [13]. The structure of these compounds varies from compact and steroid-like (fibrates) to long chain and linear (conjugated linoleic acid (CLA)).

The large number of identified coactivators has prompted the work into determining how protein complexes around nuclear receptors containing these coactivators are formed. The binding of ligand induces three-dimensional conformation changes in nuclear receptors [14, 15]. Many coactivators are recruited to nuclear receptors upon ligand binding that suggests that the conformation changes may be exposing binding sites for coactivators that are unavailable in the absence of ligand. Unique conformation changes allow for the recruitment of a specific group of coactivators in the presence of a particular ligand, thus allowing for regulation of a subset of genes in response to the ligand bound. Identifying specific coactivators that are recruited by PPARα in response to different ligands would allow for better classification of compounds and prediction of gene regulation patterns after exposure to PPs. The ability of PPARα to be activated by noncarcinogenic compounds such as CLA suggests that not all PPARα ligands are harmful. A better understanding of how conformational changes in PPARα can influence protein complex formation and ultimately affect gene regulation could lead to the development and discovery of other PPs with potentially beneficial effects on human disease.

In these studies, the ability of PPARα to recruit specific coactivators in the presence of different ligands was examined. Initial coactivator screening revealed ligand-specific coactivator recruitment. Selective inhibition of the coactivators steroid receptor, coactivator (SRC-1), PPARγ coactivator 1α (PGC-1α), and PPAR binding protein (PBP), confirmed that the ligand-induced transcriptional activity of PPAR is dependent on the recruitment of specific coactivator complexes. Limited protease digestion and peptide mapping revealed that PPARα undergoes conformational changes in response to ligands and that not all these conformations are the same. A closer examination of the PPARα amino acid sequence identified an internal LXXLL motif that can interact with other domains within the receptor, suggesting it is involved in protein folding. Mutation of this NR box resulted in a loss of ligand-induced transcriptional activity. This mutation did not cause a loss of binding to DNA or dimerization with RXRα. These results suggest that activation of PPARα is dependent on ligand-induced conformation changes allowing for the recruitment of unique transcriptional complexes around the PPAR-RXRα heterodimer.

## EXPERIMENTAL

### Materials

Wy-14,643 ([4-chloro-6-(2,3-xylindino)-2-pyrimidinylthio] acetic acid, CAS No. 50892-23-4, > 98% pure) was purchased from Chemsyn Science Laboratories (Lenexa, KS). ETYA (5, 8,11,14-Eicosatetraynoic acid), MK886, and PGJ_2_ were purchased from Biomol (Plymouth Meeting, PA). Bezafibrate, clofibric acid, ciprofibrate, dimethylsulfoxide (DMSO), and all other chemicals were obtained from Sigma (St Louis, MO). Media components were from Invitrogen (Carlsbad, CA). Fetal bovine serum (FBS) was purchased from HyClone Laboratories (Logan, UT). Components for real-time PCR were purchased from Applied Biosystems (ABI, Foster City, CA). Plasmid purification kits were purchased from Qiagen (Chatsworth, CA). All oligos were purchased from Operon (Alameda, CA). Other chemicals and reagents were of the highest grade readily available.

### Plasmids

pFR-luciferase (UAS luciferase, Gal4 response element) was purchased from BD Biosciences Clontech (Palo Alto, CA). pRL-TK Renilla (pRL-TK), pRL-CMV Renilla (pRL-CMV), pSV-β-Gal, and pGEM-T Easy were purchased from Promega (Madison, WI). The construction of pM/PPARα constructs was described previously [16]. The PPAR response element reporter, pACO(−581/−471)G.Luc was supplied by Dr Jonathon Tugwood (Zeneca Pharmaceuticals, Central Toxicology Laboratories, Maccelsfield, UK) and has been previously described [17]. The last 30 amino acids of PPARα were amplified using PCR with a 5′ BamHI and 3′ SalI restriction sites. Fragment was cloned into the pVP16 vector (Clontech, Palo Alto, CA).

### Cell culture

The COS-1 cells were maintained in α-MEM with 8% FBS and with 1% penicillin/streptomycin. 3T3L1 preadipocytes were maintained in Dulbecco's modified Eagle's medium (DMEM) with 4.5 g/L D-glucose, 10% FBS, and 1% penicillin/streptomycin.

### Transfection and reporter assays

All transient transfections were performed using LipofectAMINE (Invitrogen, Carlsbad, CA) according to the manufacturer protocol. Transfections were carried out in 24 well plates with cells plated at 50 000 cells per well and allowed to recover overnight before transfection. Peptide library was obtained as a gift from Chang et al [18]. Four hundred μg/well of GAL4DBD fusion plasmids or corresponding expression plasmid were transfected with 100 μg/well of reporter (PPRE driven or GAL4 driven) and 100 μg/well transfection efficiency control (pRL-TK). Four hundred μg/well of RNAi plasmid was used for inhibition experiments. All mammalian-2-hybrid experiments used 400 μg/well VP16AD fusion plasmid. In each experiment, the total amount of DNA transfected per well was held constant using empty vector plasmid (pcDNA3). RNA inhibitor experiments were performed in the same manner.

### Protease digestion

Full length rat PPARα was in vitro translated and labeled with S^35^-methionine and purified using Chromaspin columns (Clontech, Palo Alto, CA) to remove unincorporated radionucleotide. Purified, radiolabeled protein was concentrated using Centricon 20 concentrators (Millipore, Billerica, MA) and quantitated using a scintillation counter to ensure adequate labeling. PPARα was bound to the ligand for 30 minutes at room temperature and then digested with 50 μg/mL of α-chymotrypsin for 20 minutes at room temperature. Resulting digests were resolved on an 18% tris-glycine gel. The gel was dried and subjected to autoradiography.

### Small inhibitor RNA (RNAi) plasmids

All RNAi plasmids were made in pSUPER.neo (OligoEngine, Seattle, WA). Target sequences were chosen according to the manufacturer protocol and are listed in Table 1. Oligos were annealed and phosphorylated also according to the manufacturer protocol and cloned into linearized vector that was digested with BglII and HindIII.

### Statistical analysis

Where indicated, the MiniTab (State College, PA) was used to evaluate data for statistical significance using one-way ANOVA and Tukey's multicomparison test with significance at *P* < .05.

## RESULTS

### Identification of ligand-specific PPARα coactivators

**Figure 1:**
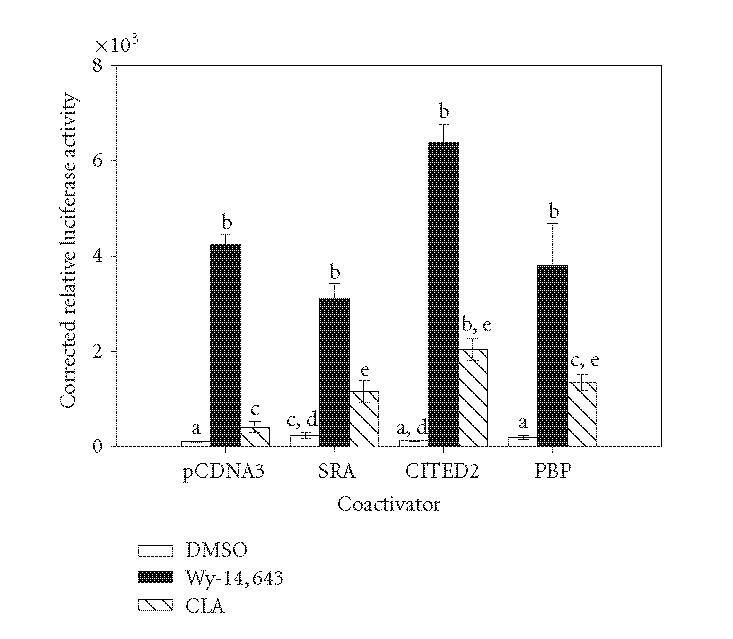
Effects of various coactivators on the activation of PPARα by Wy-14,643 and CLA. COS-1 cells were transiently transfected with pM/PPARα, GAL4 reporter, and coactivator expression plasmids or empty vector control (total DNA per well was held constant). Cells were treated for 6 hours with 50 μM Wy-14,643, 100 μM CLA, or DMSO. Luciferase activity was measured and corrected for transfection efficiency and extraction yield (*n* = 3). Relative values are corrected to untreated pcDNA3 bar (100%). Bars with different letters above are significantly different from each other (Tukey's multicomparison test, *P* < .05), representative of 2 independent experiments.

An initial screen of some known PPARα coactivators revealed differential activation in the presence of vehicle (DMSO), Wy-14,643, and CLA (Figure 1). Over expressing the RNA coactivator SRA increased the basal activity of PPARα and CLA induction, but had no effect on Wy-14,643 activity. Conversely, the CBP/p300 interacting protein CITED2 increased PPARα activity in the presence of CLA with a modest, nonsignificant increase in the presence of Wy-14,643. The LXXLL-containing coactivator PBP did not increase the activity of PPARα in the presence of either of the activators. This finding prompted a closer examination of the effect of ligand binding on the recruitment of coactivators to the PPARα transcriptional complex.

### Targeted inhibition of PPARα coactivators

**Figure 2:**
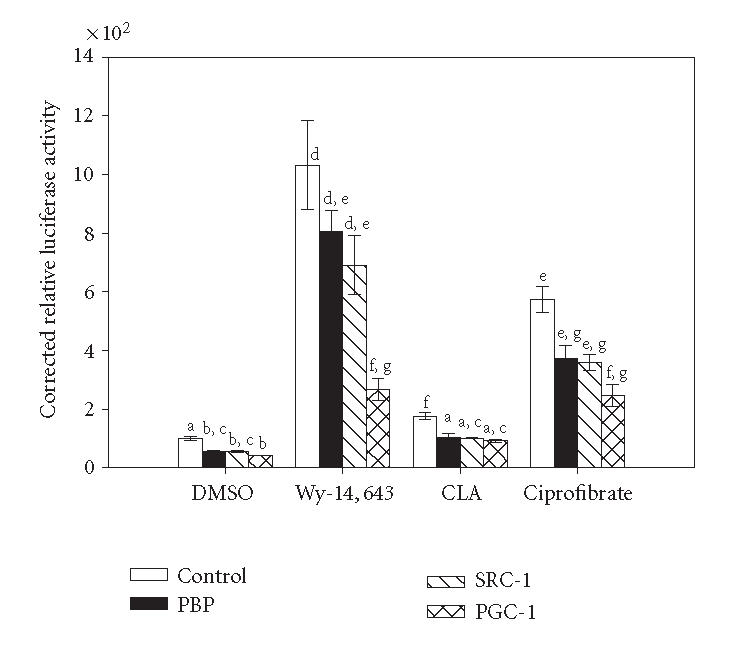
Inhibition of coactivator expression by RNAi decreases PPARα activity in a ligand-specific manner. 3T3L1 cells were transiently transfected with pM/PPARα, GAL4 responsive reporter, pRLTK, and appropriate RNAi vector or empty vector control. Cells were treated with 50 μM Wy-14,643, 100 μM CLA, 100 μM ciprofibrate, or DMSO for 6 hours. Luciferase activity was measured and corrected for transfection efficiency and extraction yield (*n* = 3). Values are expressed corrected to untreated empty vector control bar (100%). Bars with different letters above are significantly different from each other (Tukey's multicomparison test, *P* < .05), representative of 2 independent experiments.

RNA inhibition (RNAi) has become a useful tool to selectively reduce the expression of target proteins from the cellular environment. RNAi sequences were designed for three known PPARα coactivators: PBP, PGC-1α, and SRC-1. The chosen RNAi sequences were cloned into an expression plasmid that is driven by the RNAH1 promoter to generate double stranded hairpin RNA molecules. As shown in Figure 2, the basal activity of PPARα was reduced with each coactivator RNAi. Interestingly, only the inhibition of PGC-1α resulted in a significant decrease in Wy-14,643 or ciprofibrate activation of PPARα (when compared to the pcDNA3 transfected cells). Induction by CLA or ciprofibrate was lost with each coactivator RNAi. Under these conditions the expression of the three coactivators was reduced to approximately 50% of that of the control plasmid-transfected cells (data not shown).

### Examination of ligand-induced conformation changes in PPARα

**Figure 3:**
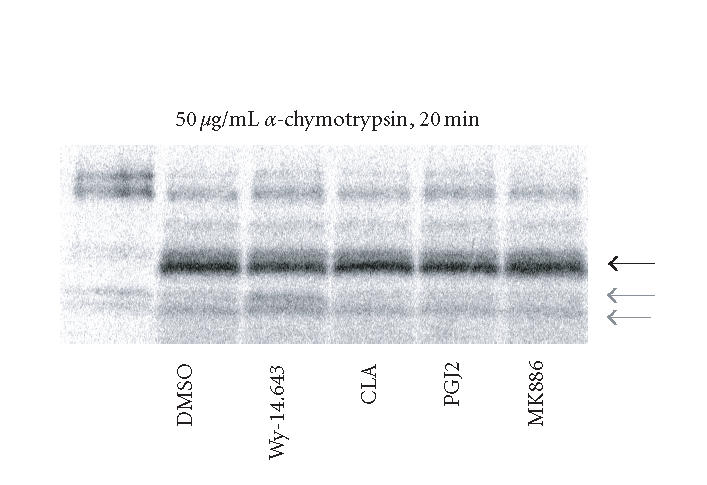
Protease digestion of in vitro translated PPARα. Rat PPARα was in vitro translated and labeled with S^35^ methionine. Unincorporated S^35^ was removed with size exclusion resin and the labeled protein was concentrated. Yield was determined and 10 fmol of PPARα were used in each digestion. PPARα was bound to the ligand for 30 minutes at 22°C and then digested with 50 μg/mL of α-chymotrypsin for 20 minutes at 22°C. Resulting digests were resolved on an 18% tris-glycine gel. The constant product is shown with the dark arrow and the Wy-14,643-protected fragments are shown with light arrows, representative of 3 independent experiments.

One possible mechanism behind the ligand-specific coactivator recruitment seen in the transient transfection and RNAi studies is the induction of unique conformational changes in PPARα. Ample evidence shows that nuclear receptors will undergo conformation changes when bound to ligand [14, 15]. To examine this phenomenon, limited protease digestion was performed using in vitro translated PPARα. As shown in Figure 3, protease digestion of Wy-14,643-activated PPARα produced a banding pattern of digestion products that was different from that of unactivated (DMSO) and CLA activated PPARα. Other compounds were tested in this manner. The PPARγ specific activator PGJ_2_ and the PPARα antagonist MK886 lead to a pattern similar to that of DMSO (data not shown). In addition, other proteases and digestion conditions were attempted with little improvement in resolution. The identification of a detectable change in the conformation of PPARα in response to a known ligand suggested that PPARα does indeed undergo conformation changes. We then sought to find a more sensitive method of evaluating these conformational changes.

**Figure 4:**
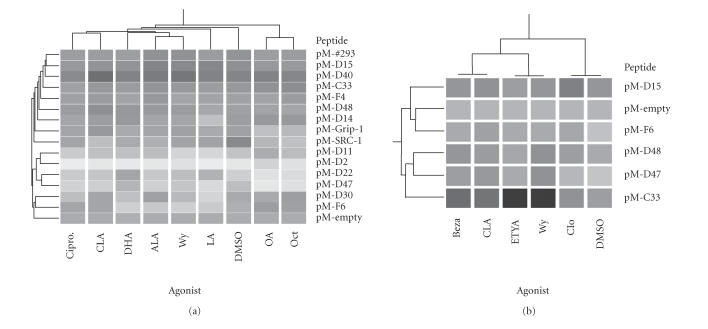
Interaction between LXXLL peptides and PPARα in mammalian two-hybrid assay. COS-1 cells were grown in serum-free media for 12 hours before transfection with GAL4DBD(pM)/peptide fusions, VP16/PPARα, pFR-luciferase, and pRL-TK. Panel (a): cells were treated with 100 μM α-linolenic acid (ALA), linoleic acid (LA), docosahexaenoic acid (DHA), conjugated linoleic acid (CLA), octanoic acid (OCT), oleic acid (OA), 25 μM Wy-14,643 (Wy), 25 μM ciprofibrate (Cipro), or DMSO (0.1% v/v) for 6 hours. Panel (b): cells were transfected and treated as described above with DMSO, CLA, Wy, and also with 100 μM eicosatetraynoic acid (ETYA), bezafibrate (Beza), or clofibrate (Clo). Luciferase activity was measured and corrected for extraction yield and transfection efficiency (*n* = 3). Data was expressed relative to that of the pM-empty construct and the presented heat map was generated in GeneSpring (Agilent, Palo Alto, CA). Darker shading represents higher luciferase activity.

Chang et al previously developed a library of randomly designed LXXLL containing peptides which were fused to the GAL4-DNA binding domain (DBD) and used to create interaction maps for the estrogen receptor (ER) [18]. This library was used to screen potential peptides that interact with PPARα using a VP16/PPARα fusion (Figure 4). Hierarchical clustering was used to compare the similarity between the interaction patterns between PPARα activated by several xenobiotic or natural ligands. Interestingly, the pattern of peptide recruitment to xenobiotic-liganded PPARα was dissimilar. Wy-14,643 was most like docohexaenoic acid (DHA) and α-linolenic acid (ALA) while ciprofibrate's interaction pattern resembled that of CLA (Panel (a)). A smaller subset of peptides was examined for a variety of xenobiotics (Panel (b)). This limited set of LXXLL peptides was able to discriminate between strong (Wy and ETYA), moderate (CLA and bezafibrate), and weak (clofibrate) agonists. These studies show the utility of the peptide libraries in mapping conformational change, as well as depicting a potential reason for differences in coactivator recruitment between Wy-14,643 and CLA as shown above.

### Role of PPARα's LXXLL motif in coactivator recruitment

**Figure 5:**
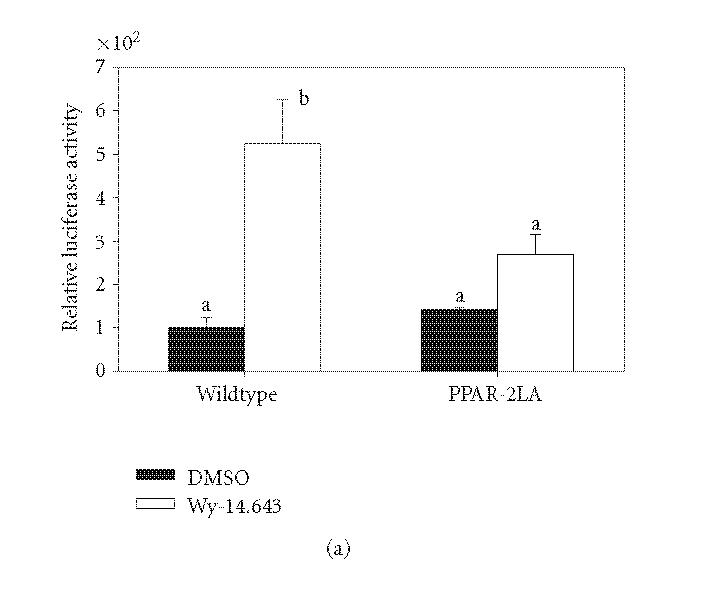

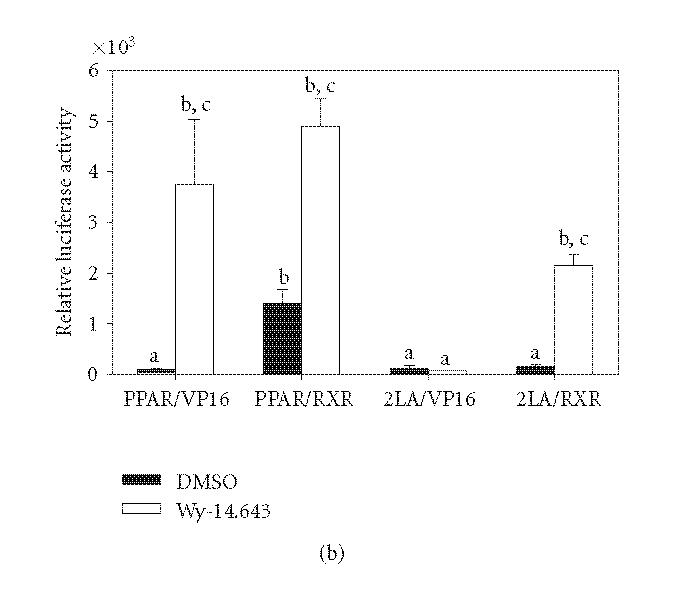

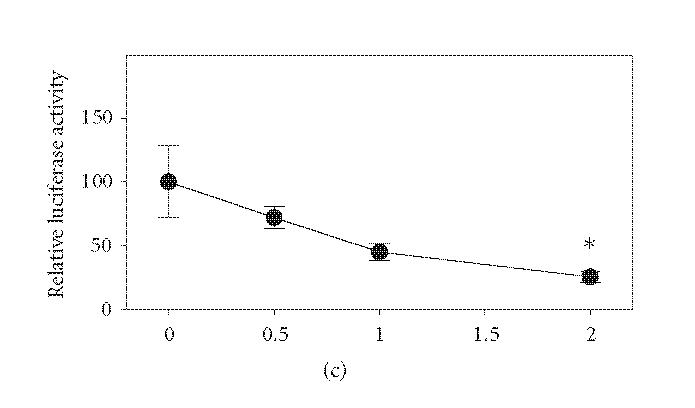
Mutation of ^456^LHPLL in PPARα decreases PP-induced transcriptional activity. Panel (a): ^456^LHPLL of rat PPARα was mutated to ^456^LHPAA (mutant designated 2LA) using site-directed mutagenesis. COS-1 cells were transiently transfected with wild type or 2LA mutant PPARα, a PPRE driven luciferase, and transfection efficiency control. Cells were treated for 6 hours with 50 μM Wy-14,643 and assayed for luciferase activity which was corrected for transfection efficiency and extraction yield (*n* = 3). Relative luciferase levels were corrected to wild type untreated bar (100%). Bars with different letters above are significantly different from each other (Tukey's multicomparison test, *P* < .05). Panel (b): COS-1 cells were transiently transfected with PPARα wild type and 2LA mutant and were fused to the GAL4DBD and transfected with VP16AD fused to the RXRα or empty vector. As noted on the axis, PPAR-VP16 is a cotransfection of pM/PPARα and empty VP16AD vector. Cells were treated with 50 μM Wy-14,643 for 6 hours. Luciferase activity was measured and corrected for transfection efficiency and extraction yield (*n* = 3). Relative luciferase levels were corrected to wild type VP16 untreated bar (100%) and plotted along a log *y*-axis to allow for ease of viewing. Bars with different letters above are significantly different from each other (Tukey's multicomparison test, *P* < .05). Panel (c): COS-1 cells were transiently transfected with a constant amount of wild type PPARα expression plasmid, a PPRE-driven reporter with increasing amounts of 2LA mutant expression plasmid. Total DNA transfected per well was held constant. Cells were untreated and assayed for luciferase activity and corrected for transfection efficiency and extraction yield (*n* = 3). Relative luciferase activity was corrected to the no mutant values (100%). *P < .05 compared to 0 μg 2LA mutant plasmid data point, representative of 2 independent experiments.

Examination of the amino acid sequence of the PPARs across all subtypes and species revealed a highly conserved LXXLL (^456^LHPLL) motif in the carboxy-terminal end of the protein in helix 12, a region of the NRs involved in conformational change and coactivator recruitment [12]. Targeted mutagenesis of ^456^LHPLL to ^456^LHPAA in full length PPARα (designated 2LA) resulted in reduced Wy-14,643-mediated response of a PPRE-driven reporter construct (Figure 5(a)). Wy-14,643 increased reporter activity nearly 6-fold in wild type PPARα versus 2-fold for the 2LA mutant. To further characterize this loss of activity, the ability of the 2LA mutant to interact with the PPARα dimer partner RXRα was examined. As shown in Figure 5(b), the wild type PPARα can interact with RXRα in the presence and absence of exogenous ligand. Wy-14,643 increased the interaction between PPARα and RXRα in the wild type PPARα by approximately 3 fold. Interestingly, the 2LA mutant was unable to interact with RXR in the absence of ligand. With the PPARα 2LA construct, Wy-14,643 increased the association with RXRα by greater than 14-fold. Thus, 2LA is able to interact with RXRα, though not in exactly the same manner as wild type. Given that the 2LA mutant is able to perform many of the functions seen with wild type PPARα but shows reduced induction by agonist, it was hypothesized that the 2LA mutant may act as a dominant negative of PPARα activity. The addition of increasing amounts of 2LA mutant with a constant amount of wild type PPARα resulted in a dose-dependent decrease in PPARα transcriptional activity (Figure 5(c)).

### Role of PPARα's LXXLL motif in intra- and intermolecular interaction

**Figure 6:**
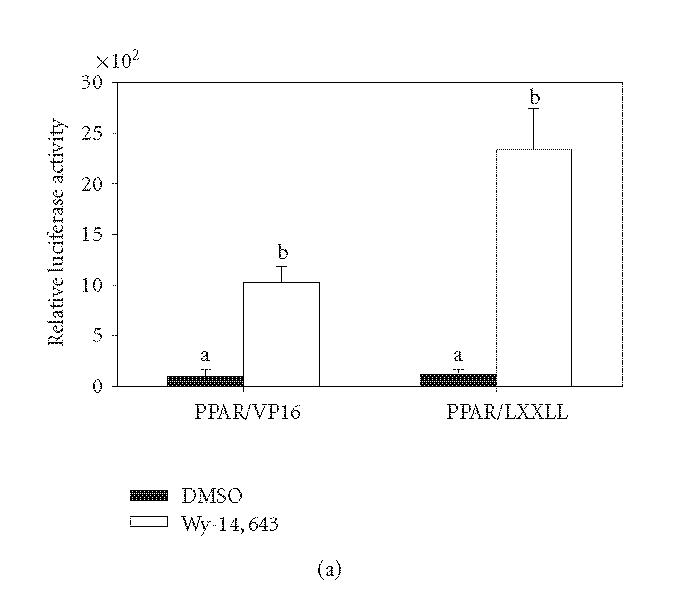

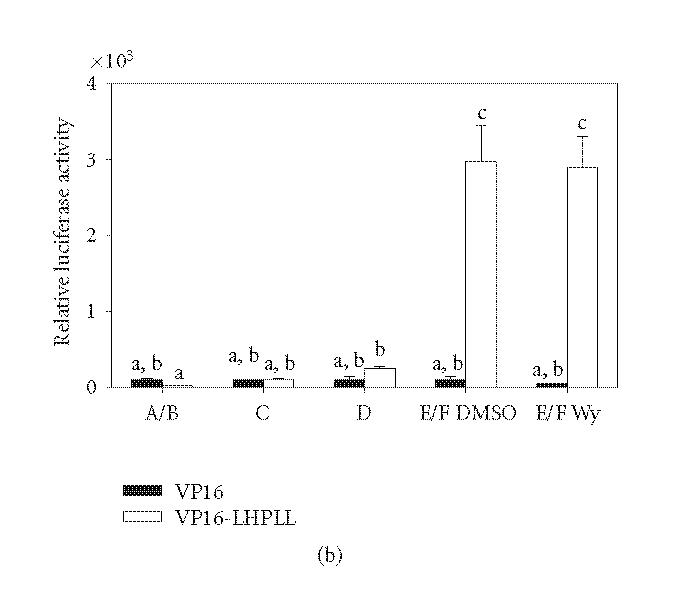

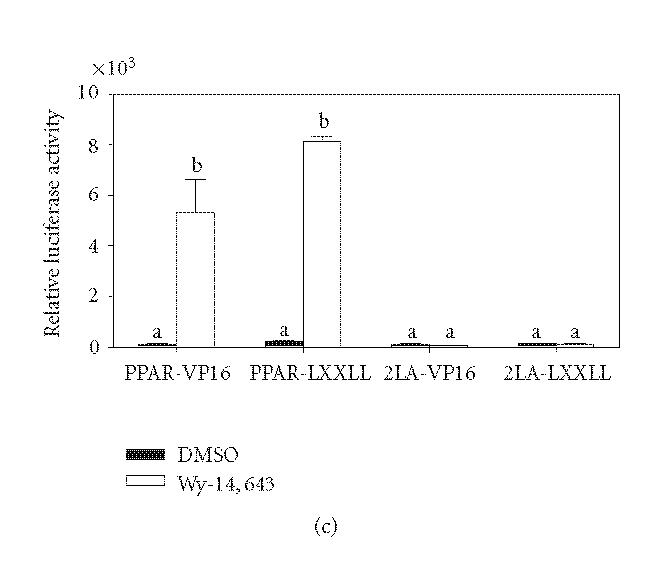
Intramolecular interaction of PPARα LXXLL motif. Panel (a): ^456^LHPLL of wild type PPARα was fused to the VP16AD and used in a mammalian-2-hybrid assay with pM/PPARα. COS-1 cells were transfected and treated with 50 μM Wy-14,643 for 6 hours. Luciferase activity was measured and corrected for transfection efficiency and extraction yield (*n* = 3). Bars are standardized to the pVP16 DMSO control bar (100%). Bars with different letters above are significantly different from each other (Tukey's multicomparison test, *P* < .05). Panel (b): COS-1 cells were transiently transfected with GAL4DBD fusions of PPARα or each domain with VP16AD-^456^LHPLL or empty vector control. Luciferase values were measured and corrected for transfection efficiency and extraction yield (*n* = 3). Bars are standardized to untreated VP16 value for each domain individually (100%). Bars with different letters above are significantly different from each other (Tukey's multicomparison test, *P* < 0.05). Panel (c): COS-1 cells were transiently transfected with PPARα wild type and 2LA mutant and were fused to the GAL4DBD and transfected with VP16AD fused to ^456^LHPLL or empty vector. Cells were treated with 50 μM Wy-14,643 for 6 hours. Luciferase activity was measured and corrected for transfection efficiency and extraction yield (*n* = 3). Relative luciferase levels were corrected to wild type VP16 untreated bar (100%). Bars are graphed on a log scale along the *y*-axis to facilitate viewing. Bars with different letters above are significantly different from each other (Tukey's multicomparison test, *P* < .05), representative of 2 independent experiments.

Since many coregulators use the LXXLL motif to interact with PPARα, we examined whether ^456^LHPLL could interact with full-length PPARα and form an intramolecular association. The last 30 amino acids of PPARα were fused to the VP16AD and used in a mammalian-2-hybrid with full-length PPARα fused to the GAL4DBD. As shown in Figure 6(a), ^456^LHPLL can interact with the full-length PPARα receptor in the presence of ligand. Mutation of ^456^LHPLL to ^456^LHPAA resulted in no interaction in the presence of ligand (data not shown). The interaction between PPARα and ^456^LHPLL was mapped using mammalian-2-hybrid. The interaction occurred between ^456^LHPLL and primarily the E/F domain with some interaction occurring with the D domain (Figure 6(b)). A repression of A/B basal activity was also seen with the addition of VP16AD-^456^LHPLL. A full-length PPARα with a mutated ^456^LHPLL (2LA) was able to interact with the PPARα ^456^LHPLL fragment in the presence or absence of ligand (Figure 6(c)) but there was still no activation by Wy-14,643. In addition, the ^456^LHPLL is able to interact with receptor interacting domains 1 and 2 (RID1 and RID2) of SRC-1 (data not shown). These results show that PPARα is able to interact with its own LXXLL motif and that the ^456^LHPLL motif in PPARα can interact with other LXXLL containing proteins.

## DISCUSSION

The recruitment of coactivators by nuclear receptor transcriptional complexes is an essential part of gene regulation through these proteins. In the presence of Wy-14,643, PBP and SRA were unable to increase the transcriptional activity of PPARα. In the presence of CLA, however, both PBP and SRA did increase the transcriptional activity of PPARα. Conversely, another known PPARα coactivator, CITED2 [19], increased PPARα activity in the presence of both CLA and Wy-14,643. These results suggested that coactivator recruitment by PPARα might depend on the identity of the ligand which is activating PPARα and that not all known coactivators of PPARα are recruited upon receptor activation. This phenomenon has been observed with PPARγ where all members of the p160 family were recruited in the presence of PGJ_2_ but none were recruited in the presence of troglitazone [20].

Selective inhibition of PPARα coactivators was achieved through the use of RNAi plasmids. Inhibition of PGC-1α resulted in a significant decrease in Wy-14,643 activation of PPARα, a loss of CLA induction, and a decrease in ciprofibrate induction. Conversely, inhibition of SRC-1 and PBP did not have a significant effect on Wy-14,643 induction but did decrease CLA and ciprofibrate induction of PPARα. The lack of significant effect upon PBP inhibition is in agreement with initial coactivator screens using overexpression of PBP in which PBP was only able to increase the activity of CLA and had no effect in the presence of Wy-14,643. In addition, analyzing the data as fold induction of luciferase activity over DMSO control for each coactivator RNAi resulted in the same conclusion. These results suggest that PGC-1α is more vital to the overall activity of the transcriptional complex than SRC-1 or PBP. While initially surprising, the ability of PPARα to overcome the inhibition of SRC-1 suggests that PPARα may be recruiting another member of the p160 family of coactivators. All three known members of the p160 family of coactivators are reasonably well conserved and the viability of the SRC-1 knockout mouse further suggests that SRC-1 may not be as essential to nuclear receptor transcriptional complexes as originally thought [21]. Currently, creation of cell lines that harbor stable inhibitions of these coactivators is being pursued. These cells lines will be used to further examine the potential impact of coactivator inhibition on gene expression and to attempt to identify genes that are coactivator-dependent as well as PPARα-dependent.

Changes in the structure of PPARα may constitute a part of the mechanism behind ligand-specific coactivator recruitment. Alterations in the three-dimensional structure of the receptor in the activated versus unactivated state is not a novel concept. Conformation changes induced by ligand binding to nuclear receptors has been previously reported for estrogen receptor and glucocorticoid receptor [14, 22, 23]. Using limited protease digestion, the binding of PPARα to Wy-14,643 induced a unique conformation compared to other known PPARα ligands suggesting that not all ligands affect PPARα 3D conformation in the same manner. The sensitivity of this technique, however, does not allow for closer examination of subtle changes in conformation. In an attempt to more closely examine the conformational changes in PPARα, a random LXXLL-containing peptide library was used. Previously, this library of LXXLL containing peptides of lengths between 19 and 22 amino acids was used to map the conformation of the estrogen receptors [18]. Our analysis showed that three compounds, Wy-14,643, bezafibrate, and CLA, induced the most similar conformation change in PPARα. Interestingly, a compound closely related to bezafibrate, clofibrate induced the most dissimilar conformation change in PPARα compared to Wy-14,643 and bezafibrate.

While clofibrate is the original compound used, clofibric acid is the active metabolic product. Both clofibrate and bezafibrate are fibrate class hypolipidemic drugs and are built upon the same phenol chemical backbone. The metabolism of clofibrate to clofibric acid appears to change the structure of the compound enough to allow PPARα to discriminate between the two. While bezafibrate and clofibrate both induce fatty acid oxidation enzymes [24], not all of the effects of these compounds are the same with regard to lipid metabolism. Bezafibrate reduces plasma apolipoprotein CIII (apoCIII) and triglyceride levels, while clofibrate does not [25]. ApoCIII is associated with the development of diabetes and a high level of apoCIII is used as a marker for hypertriglyceridemia which poses a cardiovascular risk [26, 27]. While compounds designed for the same purpose will inevitably exhibit minor differences in gene expression, the inability of clofibrate to regulate apoCIII compared to bezafibrate may be one reason that clofibrate has become a less used treatment to date. Thus, even compounds designed for the same function induce differential gene expression. This is of concern when designing novel pharmaceuticals and the mapping of the conformational changes in the PPARs may allow for the prediction of gene regulation and reveal potential unpredicted effects based on gene expression pattern.

It is clear that while the structural changes within PPARα are playing a role in ligand discrimination, the potential conformational changes that involve coactivator recruitment are less defined. A closer examination of the amino acid sequence of the PPARs revealed an LXXLL motif (^456^LHPLL) in the carboxy terminal end of all known PPARs regardless of species or subtype. Mutation of ^456^LHPLL in the full length PPARα to ^456^LHPAA (designated 2LA) results in a receptor that is unable to be transcriptionally activated by Wy-14,643 and functions as a dominant negative for PPARα. The dominant negative function of this mutation could come from either sequestration of RXRα or occupation of the PPRE without regulation of gene expression and blocking of wild type PPARα binding. Dominant negatives have been identified for coactivators like ARA54 and nuclear receptors including AR and ER [28–30] and have been used to further dissect the activity of wild type proteins. The presence of an LXXLL motif within PPARα suggests the possibility that PPARα may function as a coactivator as well. Other nuclear receptors such as SHP-1 influence gene expression without directly binding to DNA [31]. With a classic coactivator recognition sequence, other transcription complexes could use ^456^LHPLL to recruit PPARα and PPARα associated proteins.

In addition, PPARα is able to interact with its own LXXLL motif suggesting that these residues in the E/F domain are important for the conformation of PPARα. Since the binding of the PPARα LXXLL occurs within the E/F domain as shown by mammalian-2-hybrid mapping, it can be concluded that the intramolecular LXXLL interaction may prevent the binding of other LXXLL containing peptides in the absence of ligand, possibly even helping to properly form the ligand-binding pocket. Mutation of the LXXLL motif within PPARα could be disrupting the formation of ligand accepting conformation and lead to a receptor which is unresponsive to ligand activation.

The results described here show that PPARα ligands can induce unique conformation changes in the receptor and that these changes allow for the recruitment of a specific coactivator complex around the heterodimer to regulate gene expression. These conformation changes appear to involve the LXXLL motif contained within a highly conserved helix 12 region of PPARα. The fact that this LXXLL motif is conserved across all PPAR subtypes and species suggests that this motif plays a role in the overall functioning of the PPARs. A possible function of this motif could include the potential for PPARs to act as an LXXLL containing protein rather than as a pure transcription factor thus allowing PPARs to act indirectly in the regulation of gene expression, possibly as part of other protein complexes. The likelihood of this possibility, however, is unclear at this point and more work is needed to delineate this hypothesis further. Unique protein complex formation around PPARα in response to different activators reveals a mechanism behind how different ligands for PPARα can have such varied effects (carcinogen versus noncarcinogen) and how PPARα can differentiate between ligands and regulate gene expression accordingly. A better understanding of complex formation could lead to the discovery of more PPARα ligands that have beneficial effects for the prevention and treatment of human diseases.

## Figures and Tables

**Table 1:** Target sequences for coactivator RNAi.

Gene	RNAi sequence	Accession

SRC-1	5′-gttgtccgtgtaattgacc-3′	U64828
PBP	5′-gactgcctctcctatcatt-3′	NM_134027
PGC-1α	5′-agacgtccctgctcagagc-3′	NM_008904

## ABBREVIATIONS

NR: Nuclear receptor
PPAR: Peroxisome proliferator-activated receptor
PPRE: Peroxisome proliferator responsive element
PPs: Peroxisome proliferators
PBP: PPAR binding protein
PGC-1α: PPARγ coactivator 1α
SRC-1: steroid receptor coactivator-1
CITED2: CBP/p300-interacting transactivator with ED-rich tail 2
